# High-efficiency light-emitting transistors with a microcavity structure

**DOI:** 10.1093/nsr/nwaf143

**Published:** 2025-04-16

**Authors:** Chihaya Adachi

**Affiliations:** Center for Organic Photonics and Electronics Research, Faculty of Engineering, Kyushu University, Japan

Light-emitting transistors (LETs) are devices that combine the light-emitting function of organic light-emitting diodes (OLEDs) with the transistor function of controlling current [1,2]. In OLEDs, an active matrix is usually formed by installing multiple transistors outside the light-emitting component, but, in organic LETs (OLETs), this function can be achieved with a single component, so they have the advantage of a simplified device architecture [3,4]. In past devices, because OLETs shared the light-emitting layer as an OLED and the charge storage layer as a transistor, the range of material selection was quite limited and the turn-on voltage often reached several tens of volts or more. Although these devices have the potential to be applied to future organic semiconductor lasers because they can achieve ambipolar characteristics, they have many issues that need to be overcome as light-emitting devices, such as reducing the drive voltage and improving the color purity of the emitted light.

**Figure 1.**
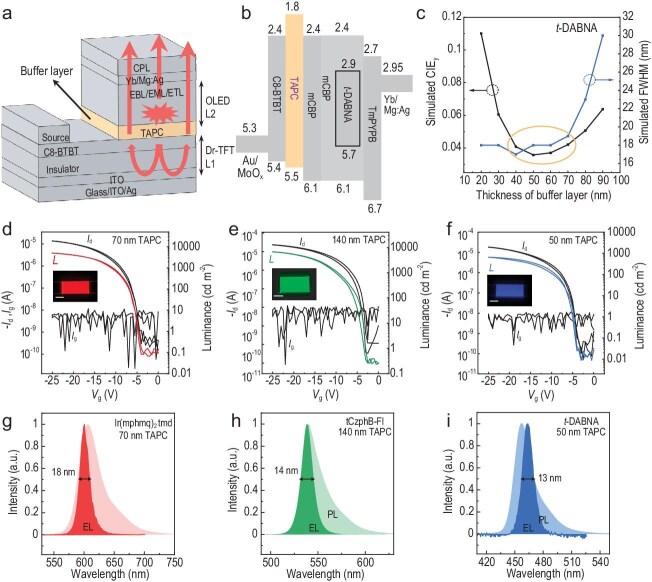
(a) Schematic illustration of the top-emitting OLET device structure with an intrinsic microcavity, in which the OLED is stacked on a TFT based on a silver substrate. TAPC serves as a buffer layer, enabling the OLET to achieve uniform area emission. CPL, capping layer; EBL, electron blocking layer; EML, emitting layer; ETL, electron transport layer; ITO, indium tin oxide. (b) Energy level diagram of the OLET based on 2,12-di-tert-butyl5,9-bis(4-(tert-butyl)phenyl)-5,9-dihydro-5,9diaza-13b-boranaphtho[3,2,1-de]anthracene (*t*-DABNA). The introduction of TAPC facilitates hole transport. (c) Commission Internationale de l'Elcairage (CIE) and full-width at half-maximum (FWHM) values as a function of TAPC thickness for the simulated spectra of the blue OLET. The optimal range of the TAPC thickness (40–70 nm) is highlighted within the ellipse. (d)–(f) Electrical and optical transfer curves of RGB-OLETs (R: red, G: green, B: blue) under specific TAPC thicknesses [70 nm for Ir(mphmq)_2_tmd-based red OLET, 140 nm for tCzphB-Fl-based green OLET, 50 nm for *t*-DABNA-based blue OLET]. (g)–(i) Comparison of the photoluminescence (PL) and EL spectra of red, green and blue emissive materials Ir(mphmq)_2_tmd, tCzphB-Fl and *t*-DABNA with intrinsically narrow emission in dilute solutions and the spectra of RGB-OLETs with microcavity effects. Reproduced with permission from [5].

In recent research published in *Nature Materials*, Professor Huanli Dong and co-workers reported on improving the performance of OLETs [5]. In this study, the researchers achieved narrowband light emission in OLETs by incorporating a microcavity-type optical resonator structure and the light-emitting and transistor functions. Differently from the conventional lateral OLETs, the developed device geometry in this study demonstrates a unique physical working mode ascribed to the innovative incorporation of a buffer layer, i.e. the 1,1-bis[(di-4-tolylamino)phenyl]cyclohexane (TAPC) layer (Fig. 1a). First, the TAPC buffer layer with a large mobility difference with

that of the thin-film transistor (TFT) conducting C8-BTBT layer effectively controls the resistance and redistributes the channel transverse electric field, thus

enabling uniform area emission, which is crucial for potential display applications. Second, the TAPC layer also acts as an effective hole-injection layer for efficient channel hole vertical transport and recombines with electrons from the cathode in the active luminescent layer for emission (Fig. 1b). In addition, the TAPC layer offers a flexible approach for tuning the microcavity effect within the architecture by adjusting its thickness, providing greater control over optical properties (Fig. 1c).

Building on this foundation, they further optimized the intrinsic microcavity, effectively narrowing the emission spectrum. By incorporating phosphorescent and thermally activated delayed fluorescence molecules into the emitting layer, the authors achieved a relatively high luminous efficiency of 20–50 cd/A and also managed to suppress the efficacy roll-off characteristics to a relatively low level (Fig. 1d–f). They narrowed the half-value bandwidth of the electroluminescence (EL) emission spectra to 18 nm for red, 14 nm for green and 13 nm for blue, resulting in the achievement of 97% of the BT2020 chromaticity (Fig. 1g–i). In addition, in terms of transistor function, by using C8-BTBT, which is a high-performance hole-transport material, the turn-on voltage was achieved at low drive voltages of 5, 3.5 and 4.5 V for blue, green and red devices, respectively. Moreover, this architecture demonstrates broad applicability and versatility, making it a promising approach for various advanced display technologies.
